# Ras, TrkB, and ShcA Protein Expression Patterns in Pediatric Brain Tumors

**DOI:** 10.3390/jcm10102219

**Published:** 2021-05-20

**Authors:** Monika Prill, Agnieszka Karkucinska-Wieckowska, Magdalena Lebiedzinska-Arciszewska, Giampaolo Morciano, Agata Charzynska, Michal Dabrowski, Maciej Pronicki, Paolo Pinton, Wieslawa Grajkowska, Mariusz R. Wieckowski

**Affiliations:** 1 Laboratory of Mitochondrial Biology and Metabolism, Nencki Institute of Experimental Biology, Polish Academy of Sciences, 02-093 Warsaw, Poland; m.prill@nencki.edu.pl (M.P.); m.lebiedzinska@nencki.edu.pl (M.L.-A.); 2 Department of Pathology, The Children’s Memorial Health Institute, 04-730 Warsaw, Poland; A.Karkucinska-Wieckowska@ipczd.pl (A.K.-W.); m.pronicki@ipczd.pl (M.P.); 3 Department of Medical Sciences, Section of Experimental Medicine, Laboratory for Technologies of Advanced Therapies (LTTA), University of Ferrara, 44121 Ferrara, Italy; mrcgpl@unife.it (G.M.); paolo.pinton@unife.it (P.P.); 4 Laboratory of Bioinformatics, Nencki Institute of Experimental Biology, 02-093 Warsaw, Poland; a.charzynska@nencki.edu.pl (A.C.); m.dabrowski@nencki.edu.pl (M.D.)

**Keywords:** pediatric brain tumors, Ras, ShcA, TrkB, malignancy grade

## Abstract

Numerous papers have reported altered expression patterns of Ras and/or ShcA proteins in different types of cancers. Their level can be potentially associated with oncogenic processes. We analyzed samples of pediatric brain tumors reflecting different groups such as choroid plexus tumors, diffuse astrocytic and oligodendroglial tumors, embryonal tumors, ependymal tumors, and other astrocytic tumors as well as tumor malignancy grade, in order to characterize the expression profile of Ras, TrkB, and three isoforms of ShcA, namely, p66Shc, p52Shc, and p46Shc proteins. The main aim of our study was to evaluate the potential correlation between the type of pediatric brain tumors, tumor malignancy grade, and the expression patterns of the investigated proteins.

## 1. Introduction

It has been previously demonstrated that Ras activity depends on ShcA recruitment upon tyrosine kinase-associated receptor (Trk) activation [1]. Abnormal action of proteins involved in this pathway may be strictly associated with oncogenic processes [2,3], especially because Ras activation has an impact on downstream oncogenes such as Raf and Rac [4,5,6], which regulate cell proliferation. The role of ShcA proteins in oncogenic processes has been intensively studied in many models, and their functions in tumors of the nervous system have gained much attention. The ShcA transcript has three isoforms, p46Shc, p52Shc, and p66Shc, which have similar structures and adaptor functions [1]. Classically, all ShcA proteins that are located in the cytoplasm and ER are involved in growth factor signaling as they become tyrosine phosphorylated in response to growth factors binding to tyrosine receptor-associated kinases at the plasma membrane [7,8]. The signal is transmitted by the ShcA complex with Grb2, thus recruits the Son of sevenless (Sos) protein, exchanging GDP for GTP in the Ras protein, which leads to its activation. Shorter ShcA isoforms (p46Shc and p52Shc) facilitate Ras activation after Grb2-Sos complex formation and binding [9]. p66Shc, the longest isoform of the Shc adaptor protein 1 subfamily, possesses an amino-terminal extension and collagen homology domain 2 (CH2), which, when phosphorylated, prevents Grb2 and Sos complex formation and therefore Ras activation [1,7,9,10]. The CH2 domain of p66Shc also contains a serine phosphorylation site that undergoes phosphorylation in response to oxidative stimuli [10]. The pro-oxidant p66Shc-associated pathway has been described as an important factor contributing to multiple pathologies by increasing oxidative stress and damage in diabetes [11], cardiovascular disorders [12], neurodegeneration [13], and mitochondrial disorders [14]. p66Shc is also an important determinant of mammalian aging [10,15] and is involved in cancer development and progression [3,16,17,18,19,20]. ShcA participation in the signal transduction pathway regulating proliferation can be potentially associated with the oncogenic process, which relies on the abnormal level or activation of plasma membrane receptors. Therefore, mutations leading to increased level or the activity of certain tyrosine kinase-associated receptors may result in constitutive Ras activation and uncontrolled proliferation [21]. Ras proteins (H-, K-, N-) are small GTPases that regulate multiple signaling pathways across membranes involved in cell adhesion, growth, migration, and survival [22], among other processes. Ras proteins are frequently mutated in different types of cancer, and the oncogenic behavior (both tumor onset and the progression of transformed cells) is given mainly by missense mutations that determine the constitutive activation of RAS downstream players [23,24]. In detail, H-RAS is the least mutated ever with a 4% probability, followed by N-RAS with 13% and finally K-RAS (83%) [25]. These three isoforms tend to have a distinguished expression among tumors; indeed, while K-RAS mutations are frequently detected in colon and pancreatic malignancies, those affecting N-RAS are found in myeloid leukemia and H-RAS in bladder cancer [26]. Interestingly, the studies of Davol et al. (2003), Frackelton et al. (2006), and Grossman (2007) showed that decreased levels of p66Shc as well as increased phosphorylation of ShcA proteins at tyrosine residues can be good markers for the diagnosis and prognosis of breast cancer and IIA colon cancer, respectively [27,28,29]. Moreover, another study showed that an increased level of p66Shc has been found in highly metastatic variants of the human breast cancer cell line MDA-MB-231 [30,31]. In neuronal cells, there are different tyrosine kinase-associated receptors, among which type A, –B, and –C can directly activate kinases such as PLCɣ1 or indirectly transmit the mitogenic signal to downstream effectors such as Ras, Raf, or extracellular regulated mitogen activated kinases (MAPK/ERK) by Shc adaptor coupling [32,33]. In 2017, a comprehensive meta-analysis of 11 studies enrolling a total of 1516 patients by Zhang C. and coworkers collected important information about tyrosine kinase-associated receptor type B (TrkB) expression found in solid tumors including gastric [34,35,36], colorectal [37,38], nonsmall cell lung [39] and ovarian [40] cancers, nasopharyngeal [41], sinonasal and oral squamous cell [42,43], and hepatocellular [40] carcinoma. Immunohistochemistry (IHC) analysis detected TrkB as overexpressed in all tumors, which was significantly associated with poor overall survival and disease-free survival of patients. Interestingly, the level of TrkB was strongly and positively associated with the clinical stage (I–II versus III–IV), classifying the protein as a potential biomarker for poor prognosis in the cohort of cancers above-mentioned [44].

In our studies, we evaluated the expression patterns of TrkB, H-, K-, and N-Ras (namely, pan-Ras) and all three isoforms of ShcA (p66Shc, p52Shc, and p46Shc) proteins in samples of brain tumors belonging to several subgroups (astrocytic, oligodendrioglial, ependymal, choroid plexus, and embryonal tumors) and defined malignancy grades (I, II, III, and IV) based on the 2016 WHO classification [45]. New prognostic markers that can facilitate and predict metastasis risk in patients are still being searched. Hence, we investigated whether the levels of Ras, TrkB, and three isoforms of ShcA, namely, p66Shc, p52Shc, and p46Shc, which may be informative in terms of pediatric brain tumor type or malignancy grade.

## 2. Materials and Methods

### 2.1. Ethics

Human studies adhered to the Declaration of Helsinki of the World Medical Association. Tumor examinations have been performed in the range of diagnostic procedures. Analyzed tissues were retrieved from the archives of the Pathology Department of the Children’s Memorial Health Institute, Warsaw, Poland, under the Bioethics Committee at the Children’s Memorial Health Institute’s approved protocol (Approval No. 155/KBE/2014 on 10 September 2014). All patients were treated according to the protocol of the Polish Pediatric Neurooncology Group. Patients gave informed consent for the use of resected samples for scientific purposes. Patient samples were anonymized.

### 2.2. Tumor Classification

Brain tumors were classified according to the World Health Organization classification of tumors of the central nervous system (CNS). The revised fourth edition of the WHO classification of tumors of the central nervous system 2016 [45] is based on a combination of histologic and molecular features for the definition of several neoplastic entities, particularly among gliomas and embryonal tumors.

### 2.3. Brain Tumor Histology and Immunohistochemistry

Forty-nine pediatric patients with pediatric brain tumors diagnosed at the Children’s Memorial Health Institute in Warsaw, Poland were included in the analysis. Analysis was performed on formalin-fixed paraffin-embedded (FFPE) and frozen tissue samples collected at diagnosis. All tumors were retrospectively reviewed according to recent WHO 2016 criteria [45].

Hematoxylin & Eosin (HE): Paraffin blocks were cut into 3-µm-thick slices and mounted on SuperFrost microscope slides. After stepwise deparaffinization and rehydration, slides were stained with hematoxylin and eosin according to standard protocols. Immunohistochemistry (IHC) was performed on the Ventana BenchMark ULTRA IHC/ISH autostaining system using mouse monoclonal and rabbit antibodies (see Table 1). After antigen retrieval in CC1 buffer, detection of the signal was followed with the Ultra View HRP system (Roche/Ventana). Whole preparations were scanned in a Hamamatsu NanoZoomer 2.0 RS scanner (Hamamatsu Photonics, Hamamatsu, Japan) at the original magnification of 40×.

The histological diagnosis of each group of brain tumors required usage of specific immunohistochemical markers, different for the individual groups of tumors. In our analysis, we used only the most common ones:

- Ki67, which is considered to be malignancy marker;
- GFAP expression allows identification of glial origin of neoplastic cells;
- Pancytokeratin expression is considered to be a marker of epithelial origin of choroid plexus tumors;
- Olig2 expression is considered to be a marker of gliomas: pilocytic astrocytoma, diffuse-type astrocytic tumors, and pediatric type of oligodendroglioma;
- INI-1 is used in the diagnosis of CNS atypical teratoid/rhabdoid tumors. INI-1 expression picture allows to distinguish atypical teratoid/rhabdoid tumor (loss of INI-1) from choroid plexus carcinoma (INI-1+);
- Synaptophysin expression allows for the identification of the neuronal origin of neoplastic cells;
- EMA staining might serve as sensitive and specific markers of ependymal differentiation in glial tumors; and
- S100 is a characteristic marker for glial tumors as well as choroid plexus tumors.

Proliferation activity of neoplastic cells was described as a Ki-67 labeling index (Ki-67 LI). Percentage of nuclear expression of Ki-67 was counted in 300 hot-spot neoplastic cells in high power microscopic fields (400×).

Cytoplasmic expression of glial fibrillary acid protein (GFAP) was assessed semi-quantitatively as negative (−), low (+), moderate (++), and strong (+++), depending on the percentage of positive cells in microscopic examination.

### 2.4. Western Blot Analysis

Brain tumors after retrieval were stored in liquid nitrogen upon sample preparation. Small fragments of pediatric brain tumors were cut with a scalpel and added to 50 μL of homogenization buffer (75 mM saccharose, 225 mM mannitol, 5 mM Tris HCl) containing protease inhibitor cocktail (1.04 mM AEBSF, 0.8 μM aprotinin, 0.04 mM bestatin, 0.14 mM E-64, 0.02 mM leupeptin, 0.015 mM pepstatin A) and phosphatase inhibitor cocktail 3 (1 mM Na3VO4 and 10 mM NaF) used at a 1:100 dilution. Then, the samples were gently homogenized in a precooled Potter Elvehjem Tissue Homogenizer. Afterward, a double portion of cold lysis buffer (50 mM Tris, 150 mM NaCl, 1% Triton X-100, 0.1% SDS, 1% sodium deoxycholate, protease inhibitor cocktail: 1.04 mM AEBSF, 0.8 μM aprotinin, 0.04 mM bestatin, 0.14 mM E-64, 0.02 mM leupeptin, 0.015 mM pepstatin A and phosphatase inhibitor cocktail 3 used at a 1:100 dilution) was added. After 30 min of incubation on ice, samples were centrifuged at 14,000× *g* for 20 min at 4 °C to remove insoluble tissue and cellular debris. After centrifugation, the supernatant was collected, and the protein concentration was determined using the Bradford method. Samples dedicated for SDS-PAGE were denatured in reducing Laemmli loading buffer at 95 °C for 5 min. Due to the technical limitations to perform electrophoretic separation of 49 samples contemporary on one acrylamide gel, 49 samples were divided into smaller groups and several western blots preceded contemporarily. Each individual gel (group of samples) contained physically the same internal control sample (reference sample, Ref) that allowed us to later compare and merge the results from the individual blots. Proteins were separated on a 4–15% gradient or 8% gel prior to ShcA and TrkB detection and 12% prior to Ras detection. Proteins were then transferred onto PVDF membranes for 90 min using a 300 mA constant current, after which the membrane was blocked using a Li-Cor dedicated TBS-based blocking buffer (Li-Cor, Odyssey). ShcA proteins p46Shc, p52Shc, and p66Shc were detected using mouse monoclonal antibodies (BD Biosciences) concentrated 1:1000 in Li-Cor blocking buffer in TBS-Tween (TBS-T). For H-, K-, and N-Ras (pan-Ras) and TrkB protein detection, mouse monoclonal antibodies from Merck-Millipore and rabbit polyclonal antibodies from Cell Signaling, respectively, were concentrated at 1:1000 in Li-Cor TBS-T buffer. The membranes were incubated overnight at 4 °C with primary antibodies. Proper anti-mouse or anti-rabbit secondary fluorescent antibodies from Life Technologies were concentrated 1:5000 in Li-Cor TBS-T blocking buffer supplemented with 0.01% SDS. The fluorescence was detected with the use of an Odyssey Infrared Imaging System (Li-Cor Biosciences, Lincoln, NE, USA) and quantified with Image Studio Lite software from Li-Cor.

Equal protein loading was verified with the use of the Revert™ total protein stain kit from Li-Cor (before incubation with primary antibodies) by whole membrane staining, and the signal was scanned with the use of a Li-Cor Odyssey scanner and quantified with Image Studio Lite software from Li-Cor. In the case of ShcA and Ras proteins, β-actin was used as a loading marker. In the case of the TrkB protein, total protein staining (Revert™ 700 Total Protein Stain, Li-Cor) was used for western blot normalization.

### 2.5. Statistical Analysis

The data were analyzed to check whether there were significant differences among tumor grades or tumor types. To compare the differences in mean values between grade- or tumor type-based groups according to measured markers, we first standardized the data. For each technical replicate of each marker, the data were first divided by the average value for this replicate. Then, each data point was averaged over all replicates for a marker. Based on this standardization, for each marker separately, we performed one-way ANOVA statistical tests, separately for tumor type and tumor WHO grades. For post-hoc multiple comparisons, we used the t-tests with Bonferroni correction. We considered as significant changes with one-way ANOVA *p* < 0.05 and post-hoc *p* < 0.05. Moreover, for directed comparison of each pair of groups for different grades, the nonparametric Wilcoxon rank test was performed. We also performed the Kruskal–Wallis rank sum test, which is the nonparametric analog of one-way ANOVA, followed by Tukey and Kramer (Nemenyi) rank tests as nonparametric post-hoc tests.

## 3. Results

### 3.1. Histopathological and Immunohistochemical Studies

To evaluate the potential correlation between the type of pediatric brain tumor (and tumor malignancy grade) and the expression patterns of Ras and ShcA proteins, we analyzed 49 samples of pediatric brain tumors. In our studies, five main groups of investigated tumors were distinguished: choroid plexus tumors, diffuse astrocytic and oligodendroglial tumors, embryonal tumors, ependymal tumors, and other astrocytic tumors. Correct assignment to the individual group was confirmed based on histopathological and immunohistochemical investigations. Supplementary Table S1 contains information about tumor type, WHO grade, localization, age, year of diagnosis, and gender of the pediatric brain tumor donors used in the studies.

Group 1 represents choroid plexus tumors. In this group, based on histopathological investigation, we distinguished two types of choroid plexus tumors: (a) choroid plexus papilloma (CPP) and (b) choroid plexus carcinoma (CPC).

**Figure 1:**
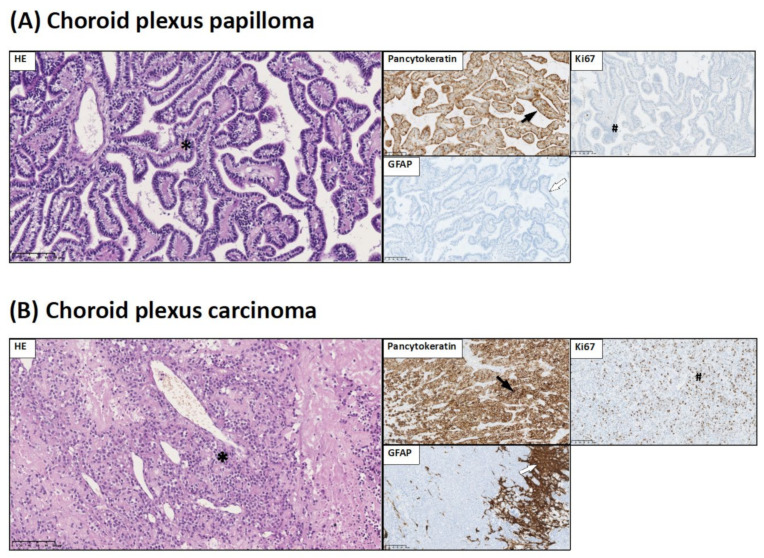
Histological and immunohistochemical staining of group 1 pediatric brain tumors: choroid plexus papilloma and choroid plexus carcinoma. HE staining as well as GFAP, Ki67, and pancytokeratin immunohistochemical staining were performed to visualize typical manifestations in these types of tumor tissue. (**A**) Choroid plexus papilloma (CPP): papillary pattern of CPP in HE staining (*). Neoplastic cells are positive for pancytokeratin immunostaining (black arrow). Low Ki67 LI in tumor tissue (#). GFAP negative staining in neoplastic cells (white arrow). (**B**) Choroid plexus carcinoma (CPC): solid pattern of CPC in HE staining (*). Neoplastic cells are positive for pancytokeratin immunostaining (black arrow). High Ki67 LI in tumor tissue (#). GFAP reactivity only in the parenchyma (white arrow). Preparations were scanned at original magnification 40×, and digital magnification presented on the image was 20× for HE and 10× for immunohistochemical preparations.

(a) Choroid plexus papilloma (WHO grade I)—a papillary neoplasm arising from choroid plexus epithelium that corresponds to WHO grade I. Histopathology of CPP showed numerous fibrovascular papillary structures covered by a single layer of cuboidal to columnar epithelium. Degenerative changes such as calcifications and hyalinization were seen (Figure 1A).

(b) Choroid plexus carcinoma (WHO grade III)—histopathological analysis revealed solid hypercellular sheets of pleomorphic epithelioid cells with brisk mitoses. In one case, foci of papillary architecture were retained. In all cases, foci of necrosis were present.

Immunohistochemistry showed that neoplastic cells of both CPP and CPC were positive for pancytokeratin and negative for GFAP (Figure 1A,B).

**Figure 2:**
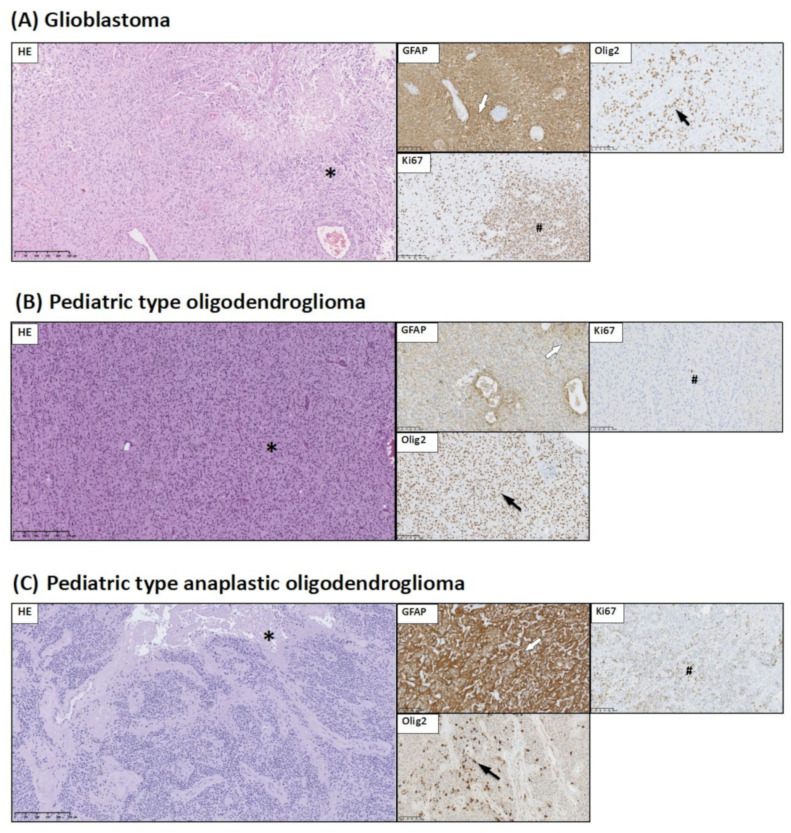
Histological and immunohistochemical staining of group 2 pediatric brain tumors: glioblastoma, pediatric-type oligodendroglioma, and pediatric-type anaplastic oligodendroglioma. HE staining as well as GFAP, Ki67, and Olig2 immunohistochemical staining were performed to visualize typical manifestations in these types of tumor tissue. (**A**) Glioblastoma: foci of micronecrosis in HE staining (*). GFAP immunoreactivity in neoplastic cells (white arrow). Olig2 immunoreactivity in neoplastic cells (black arrow). High Ki67 LI in tumor tissue (#). (**B**) Pediatric type oligodendroglioma: round neoplastic cells with a halo pattern in HE staining (*). GFAP immunoreactivity in some neoplastic cells and in parenchyma (white arrow). Olig2 immunoreactivity in neoplastic cells (black arrow). Low Ki67 LI in tumor tissue (#). (**C**) Pediatric type anaplastic oligodendroglioma: foci of necrosis in HE staining (*). GFAP immunoreactivity in neoplastic cells (white arrow). Olig2 immunoreactivity in neoplastic cells (black arrow). High Ki67 LI in tumor tissue (#). Preparations were scanned at original magnification 40×, and digital magnification presented on the image is 10× for HE and 5× for immunohistochemical preparations.

Group 2 represents diffuse astrocytic and oligodendroglial tumors. In this group, based on immunohistochemistry and histopathological investigation, we distinguished three types of diffuse astrocytic and oligodendroglial tumors: (a) glioblastoma (GB), (b) pediatric-type oligodendroglioma, and (c) pediatric-type anaplastic oligodendroglioma (Figure 2).

(a) Glioblastomas, (WHO grade IV)—a diffusely infiltrating high-grade glioma with predominantly astrocytic differentiation were characterized by foci of high cellularity, marked cellular pleomorphism, brisk mitotic activity, and microvascular proliferation. In all cases of GB, foci of ischemic necrosis were present. In one case, the palisading necrosis was seen. Immunohistochemistry of the neoplastic cells showed the expression of GFAP and Olig2. The Ki67 LI was about 50% (Figure 2A).

(b) and (c); Pediatric-type oligodendrogliomas (b) corresponded to WHO grade II and pediatric-type anaplastic oligodendroglioma (c) to WHO grade III. Oligodendrogliomas were composed of monomorphic cells with uniform round nuclei with surrounding perinuclear clearing “haloes”. The network of delicate branching capillaries resembling “chicken wire” were present in all cases. Immunohistochemistry showed that neoplastic cells were positive for Olig2, and in some cases, revealed moderate positivity for GFAP (Figure 2B,C). In anaplastic cases of oligodendroglioma, high mitotic activity and microvascular proliferation were seen. In two cases, foci of necrosis were present.

**Figure 3:**
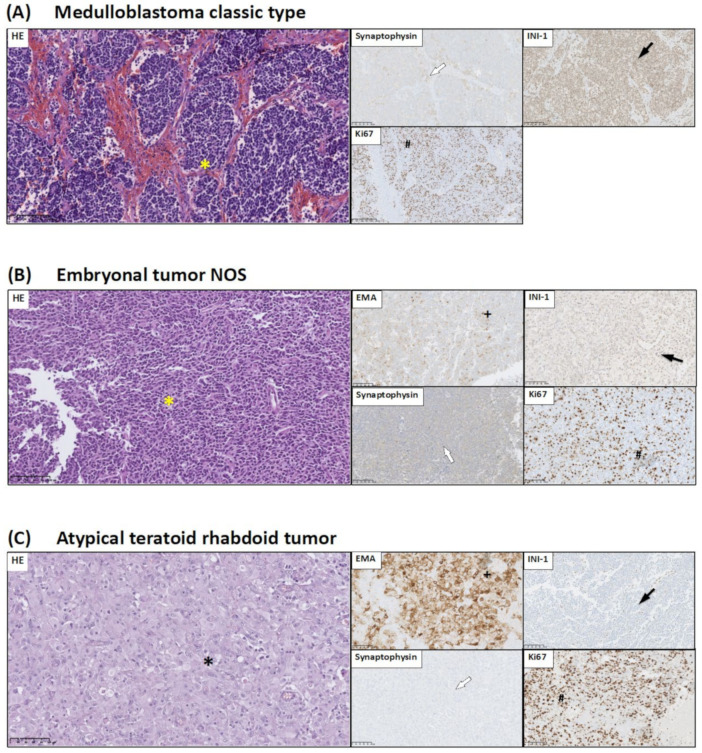
Histological and immunohistochemical staining of group 3 pediatric brain tumors: medulloblastoma classic type, embryonal tumor NOS, and AT/RT. HE staining as well as synaptophysin, Ki67, INI-1, and EMA immunohistochemical staining were performed to visualize typical manifestations in these types of tumor tissue. The picture shows the typical staining of a representative sample from each type of tumor. (**A**) Medulloblastoma classic type: small round neoplastic cells with scant cytoplasm (*). Neoplastic cells positive for synaptophysin immunostaining (white arrow). Immunoexpression of INI-1 in neoplastic cells (black arrow). High Ki67 LI in tumor tissue (#). (**B**) Embryonal tumor NOS: primitive neoplastic cells (*). Neoplastic cells with dot-like reaction of EMA (+). INI-1 immunostaining in some neoplastic cells (black arrow). Weak reaction for synaptophysin (white arrow). High Ki67 LI in tumor tissue (#). (**C**) AT/RT: rhabdoid cells in HE staining (*). Neoplastic cells strong positive for EMA immunostaining (+). Lack of INI-1 immunoexpression in neoplastic cells (black arrow). Weak reaction for synaptophysin (white arrow). High Ki67 LI in tumor tissue (#). Preparations were scanned at original magnification 40×, and digital magnification presented on image is 20× for HE and 5× for immunohistochemical preparations.

Group 3 represents CNS embryonal tumors. In this group, we distinguished three types of embryonal tumors: (a) medulloblastoma (MB), (b) embryonal tumors not otherwise specified (NOS), and (c) atypical teratoid/rhabdoid tumors (AT/RT) (Figure 3).

(a) Medulloblastoma—this embryonal tumor arises in the cerebellum or dorsal brain stem mainly in children and corresponds to WHO grade IV. All MBs were classified as classic types. They consisted of densely packed small round undifferentiated cells with high mitotic activity (Figure 3A). In three cases, Homer Wright rosettes were seen. MBs demonstrated immunoreactivity for synaptophysin. The Ki67 LI was about 50% (Figure 3A).

(b) Embryonal tumors not otherwise specified—all tumors in this group are WHO grade IV by definition. Microscopically, CNS embryonal tumors were composed of small cells with little perinuclear cytoplasm and hyperchromatic nuclei. Mitoses were abundant, and necrosis was prominent. Immunohistochemistry showed that these tumor cells were positive for synaptophysin. The Ki67 LI was 70% (Figure 3B).

(c) Atypical teratoid/rhabdoid tumor—AT/RTs were composed of undifferentiated, embryonal-like cells and foci of rhabdoid cells with round eccentric nuclei and prominent nucleoli. High mitotic activity was seen. In all tumors, necrosis was prominent. All AT/RT tumors showed focal immunoreactivity for epithelial membrane antigen (EMA) and synaptophysin. The diagnostic hallmark was the loss of INI-1 protein expression in neoplastic cells (Figure 3C).

**Figure 4:**
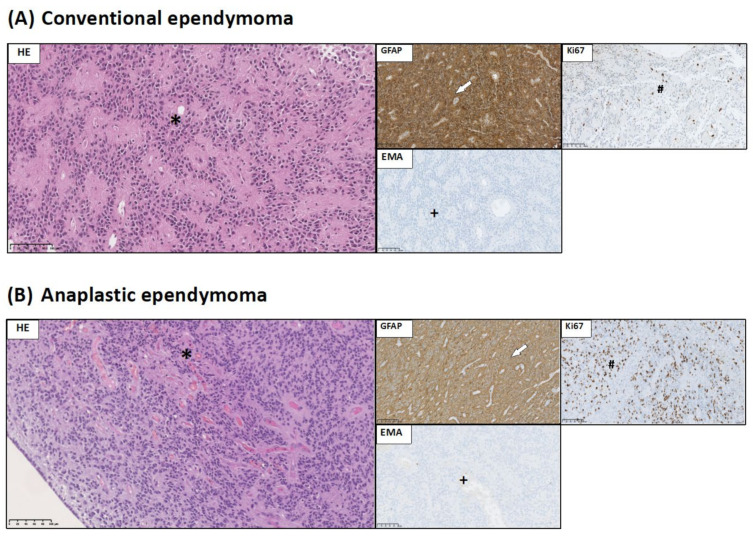
Histological and immunohistochemical staining of group 4 pediatric brain tumors: ependymoma and anaplastic ependymoma. HE staining as well as GFAP, Ki67, and EMA immunohistochemical staining were performed to visualize typical manifestations in these types of tumor tissue. (**A**) Ependymoma: pseudorosette structures (*). GFAP immunoreactivity in neoplastic cells (white arrow). EMA dot-like reaction in tumor cells (+). Low Ki67 LI in tumor tissue (#). (**B**) Anaplastic ependymoma: pseudorosette structures and microvessel proliferation (*). GFAP immunoreactivity in neoplastic cells (white arrow). EMA reaction in single tumor cells (+). High Ki67 LI in tumor tissue (#). Preparations were scanned at original magnification 40×, and digital magnification presented on image is 20× for HE and 5× for immunohistochemical preparations.

Group 4 represents ependymal tumors. In the range of this group, we distinguished two types of ependymal tumors: (a) conventional ependymoma and (b) anaplastic ependymoma (Figure 4).

(a) Conventional ependymomas—on histologic examination, these were well circumscribed, moderately cellular tumors with uniform cells. In two cases, ependymal rosettes were seen. All cases presented pseudorosettes, with perivascular tumor cells extending radial, fibrillary processes toward the vessel wall. Mitoses were rare, while necrosis was observed in two cases (Figure 4A). All samples of conventional ependymoma corresponded to WHO grade II malignancy.

(b) Anaplastic ependymoma—these were characterized by high cellular density, high mitotic activity, and a high nuclear to cytoplasmic ratio. Pseudorosettes were less prominent. Microvascular proliferations were present. Immunohistochemistry showed that ependymal tumors were positive for GFAP (Figure 4A,B). The picture of epithelial membrane antigen (EMA) expression showed a characteristic punctate, dot-like pattern of cytoplasmic positivity in neoplastic cells (Figure 4A,B).

**Figure 5:**
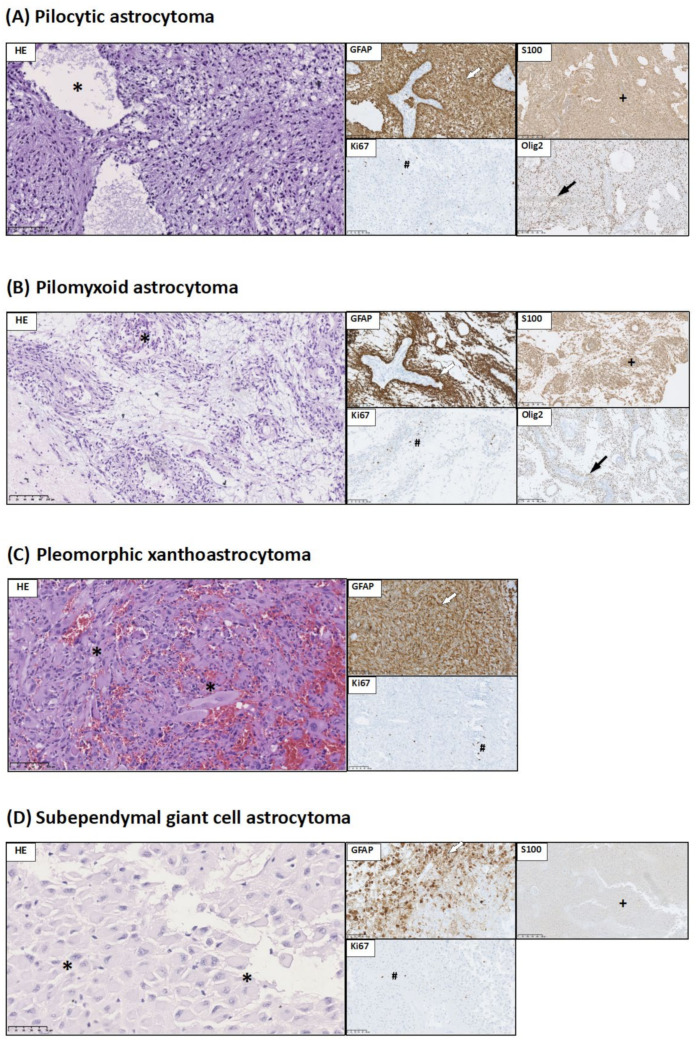
Histological and immunohistochemical staining of group 5 pediatric brain tumors: pilocytic astrocytoma, pilomyxoid astrocytoma, pleomorphic xanthoastrocytoma (PXA), and subependymal giant cell astrocytoma (SEGA). HE staining as well as GFAP immunohistochemical staining and Ki67 were performed to visualize typical manifestations in these types of tumor tissue. (**A**) Pilocytic astrocytoma: microcystic pattern (*). GFAP strong positive staining in tumor tissue (white arrow). S100 immunoreactivity in neoplastic cells (+). Low Ki67 LI in tumor tissue (#). Olig2 immunoreactivity in neoplastic cells (black arrow) (**B**) Pilomyxoid astrocytoma: myxoid changes in stroma and angiocentric pattern (*). GFAP strong positive staining in tumor tissue (white arrow). S100 immunoreactivity in neoplastic cells (+). Low Ki67 LI in tumor tissue (#). Olig2 immunoreactivity in neoplastic cells (black arrow). (**C**) PXA: pleomorphic cells (*). GFAP-positive staining in tumor tissue (white arrow). Low Ki67 LI in neoplastic cells (#). (**D**) SEGA: ganglion-like cells (*). GFAP-positive staining in tumor tissue (white arrow). Low Ki67 LI in neoplastic cells (#). Weak of S100 immunoreactivity in neoplastic cells (+). Preparations were scanned at original magnification 40×, and digital magnification presented on image is 20× for HE and 5× for immunohistochemical preparations.

Group 5 represents tumors characterized by the WHO as other astrocytic tumors. In the range of this group, we distinguished four types of tumors: (a) pilocytic astrocytoma, (b) pilomyxoid astrocytoma, (c) pleomorphic xanthoastrocytoma (PXA), and (d) subependymal giant cell astrocytoma (SEGA) (Figure 5).

(a) Pilocytic astrocytoma—on histologic examination, these were characterized by low to moderate cellularity and a biphasic growth pattern, consisting of compact areas with bipolar (piloid) tumor cells and microcystic areas with multipolar tumor cells. Rosenthal fibers and eosinophilic granular bodies were seen. Microvascular proliferation and degenerative cellular pleomorphism were observed. This type of tumor demonstrates strong immunoreactivity for GFAP, S100, and Olig2 (Figure 5A).

(b) Pilomyxoid astrocytoma—on histologic examination, it showed that tumor cells form pseudorosette-like angiocentric architectures. Interestingly, intermediate forms between pilocytic and pilomyxoid astrocytoma have also been reported. Tumor cells are strongly immunoreactive for a glial fibrillary acid protein (GFAP), Olig2, and S100. The Ki67 LI was low, less than 3% (Figure 5B).

(c) Pleomorphic xanthoastrocytoma (PXA)—histologic features of PXA included the presence of pleomorphic, sometimes bizarre, and multinucleated giant cells, lipidized astrocytic tumor cells, eosinophilic granular bodies, often perivascular lymphocytic infiltrates, and a variably dense pericellular/perilobular network of reticulin fibers. A fascicular growth pattern was often observed. GFAP immunoreactivity in neoplastic cells was strong. The Ki67 LI was less than 3% (Figure 5C).

(d) Subependymal giant cell astrocytoma (SEGA)—histopathological examination showed that SEGAs are moderately cellular tumors composed of pleomorphic large astrocytic or ganglioid cells with abundant glassy eosinophilic cytoplasm and round, vesicular nuclei with distinct nucleoli. In some cases, smaller spindle cells arranged in streams were commonly encountered. Multinucleated cells were present in two cases. The formation of perivascular pseudorosettes mimicking ependymal pseudorosettes was also seen. The presence of necrotic areas was observed in single cases. Most SEGA tumor cells presented immunoreactivity for GFAP and S100. The Ki67 LI was about 2% (Figure 5D).

Semiquantitative assessment of cytoplasmic expression of GFAP as negative (−), low (+), moderate (++), and strong (+++) as well as the Ki-67 labeling index (Ki-67 LI) describing the proliferation activity of neoplastic cells are presented in Supplementary Table S2. The expression profile of Ki67 expressed as Ki-67 LI strongly correlates with the grade of brain tumors (Supplementary Table S2).

**Figure 6:**
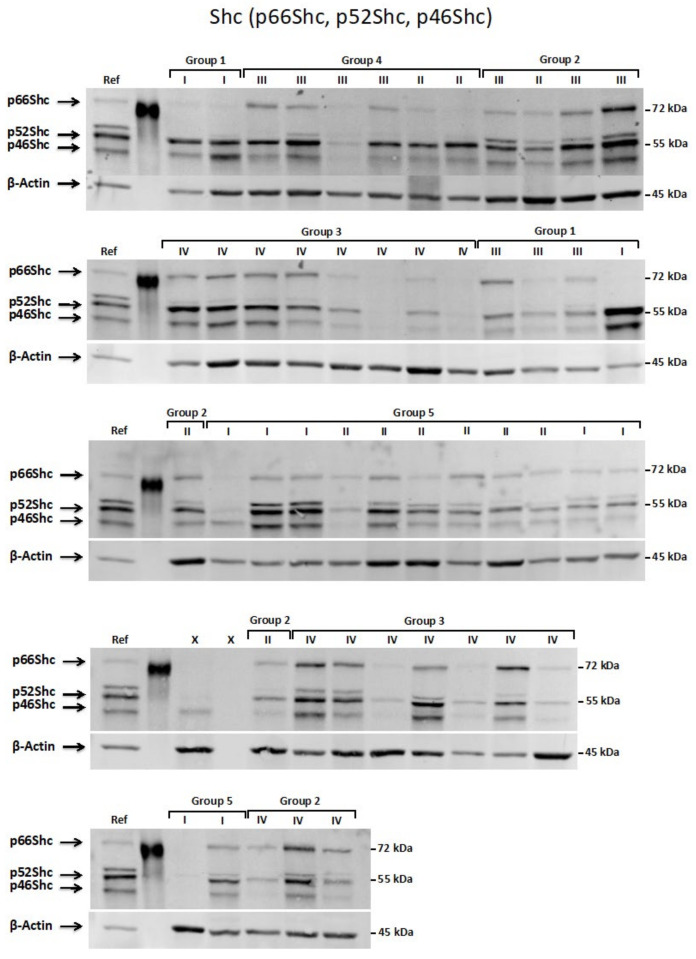
Western blot analysis of ShcA isoform levels in investigated pediatric brain tumors. The figure shows representative image of p66Shc, p52Shc, and p46Shc levels. The assignment to the tumor type and tumor grade is shown above the individual samples. Group 1—choroid plexus tumors; Group 2—diffuse astrocytic and oligodendroglial tumors; Group 3—embryonal tumors; Group 4—ependymal tumors; Group 5—other astrocytic tumors. Roman numerals (I, II, III, and IV) represent the grade of malignancy based on the WHO grade, representing the severity and prognosis of the condition. Reference sample (Ref). The level of p66Shc, p52Shc, and p46Shc was assessed immunochemically with the use of specific antibodies and subjected to the statistical analysis described in the Materials and Methods section.

**Figure 7:**
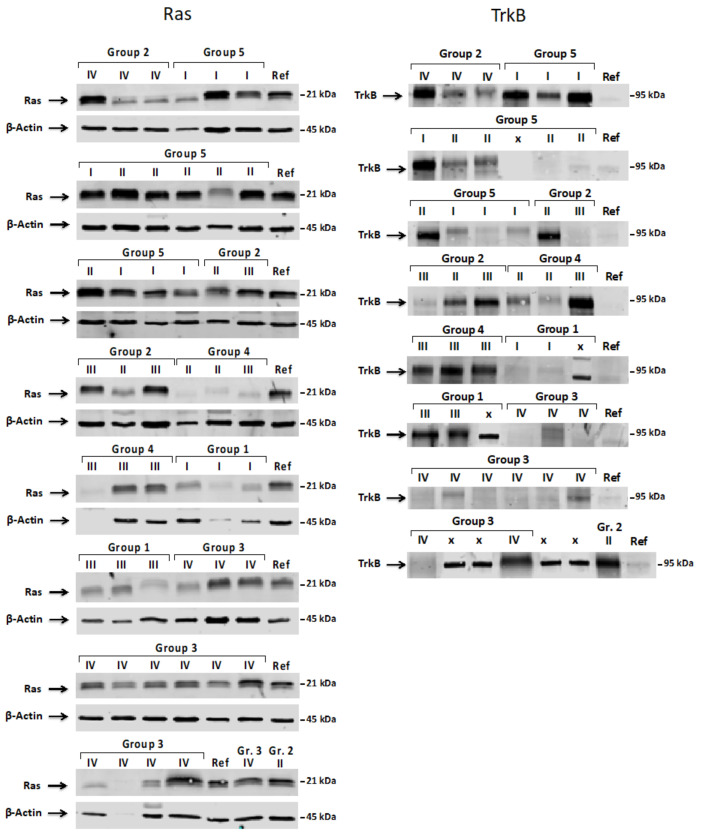
Western blot analysis of Ras and TrkB proteins levels in investigated pediatric brain tumors. The figure shows a representative image of the Ras and TrkB levels. The assignment to the tumor type and tumor grade is shown above the individual samples. Group 1—choroid plexus tumors; Group 2—diffuse astrocytic and oligodendroglial tumors; Group 3—embryonal tumors; Group 4—ependymal tumors; Group 5—other astrocytic tumors. Roman numerals (I, II, III, and IV) represent the grade of malignancy based on the WHO grade, representing the severity and prognosis of the condition. Reference sample (Ref). The level of Ras and TrkB was assessed immunochemically with the use of specific antibodies and subjected to the statistical analysis described in the Materials and Methods section.

Next, when the assignment of individual samples to the individual group was confirmed, we evaluated the expression patterns of ShcA isoforms (p66, p52 and p46) as well as Ras and TrkB proteins in all characterized tumors samples. Figure 6 and Figure 7 show representative images of p66Shc, p52Shc, p46Shc, Ras, and TrkB levels in the investigated tumor samples.

### 3.2. Expression Pattern of ShcA, Ras and TrkB Proteins in Pediatric Brain Tumors and Their Levels in a Function of Tumor Malignancy Grade

**Figure 8:**
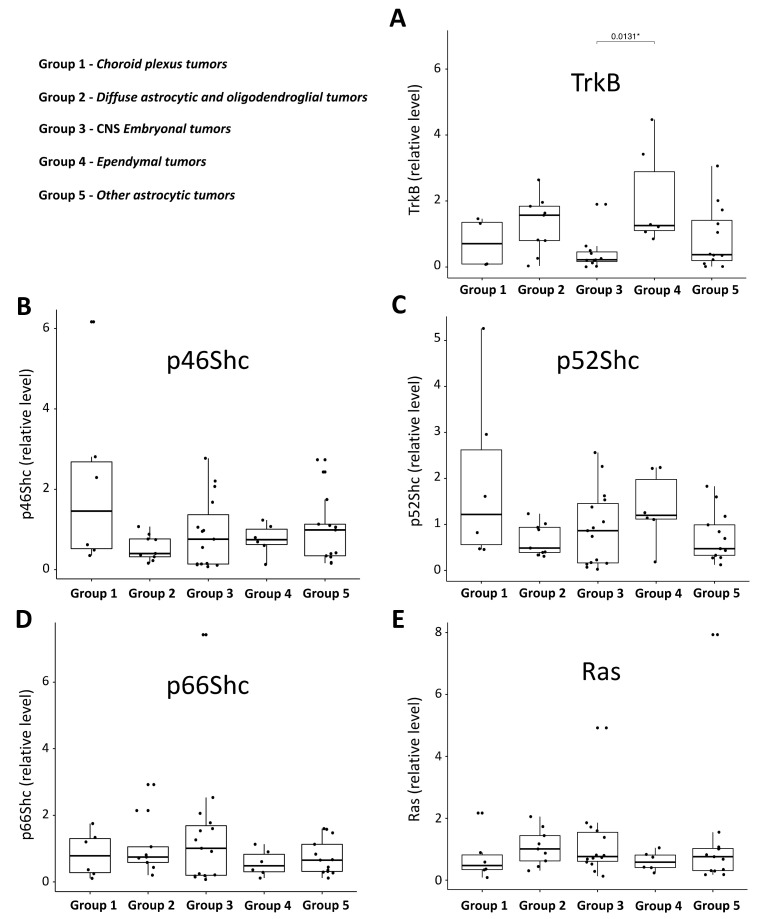
Box plot graphs showing the TrkB, Ras, and ShcA isoforms’ western blot quantification in investigated pediatric brain tumors. The presented box plots show the standardized levels of TrkB (**A**), p66Shc (**D**), p52Shc (**C**), p46Shc (**B**), and Ras (**E**) proteins in the five groups of tumor samples divided based on the histochemical analysis presented in Figure 1, Figure 2, Figure 3, Figure 4 and Figure 5 and summarized in Supplementary Tables S1 and S2. The position of each dot on the vertical axis indicates values for an individual data point. * *p* < 0.05. The level of TrkB, p66Shc, p52Shc, p46Shc, and Ras was assessed by the western blot technique with the use of specific antibodies and subjected to the statistical analysis described in the Materials and Methods Section.

**Figure 9:**
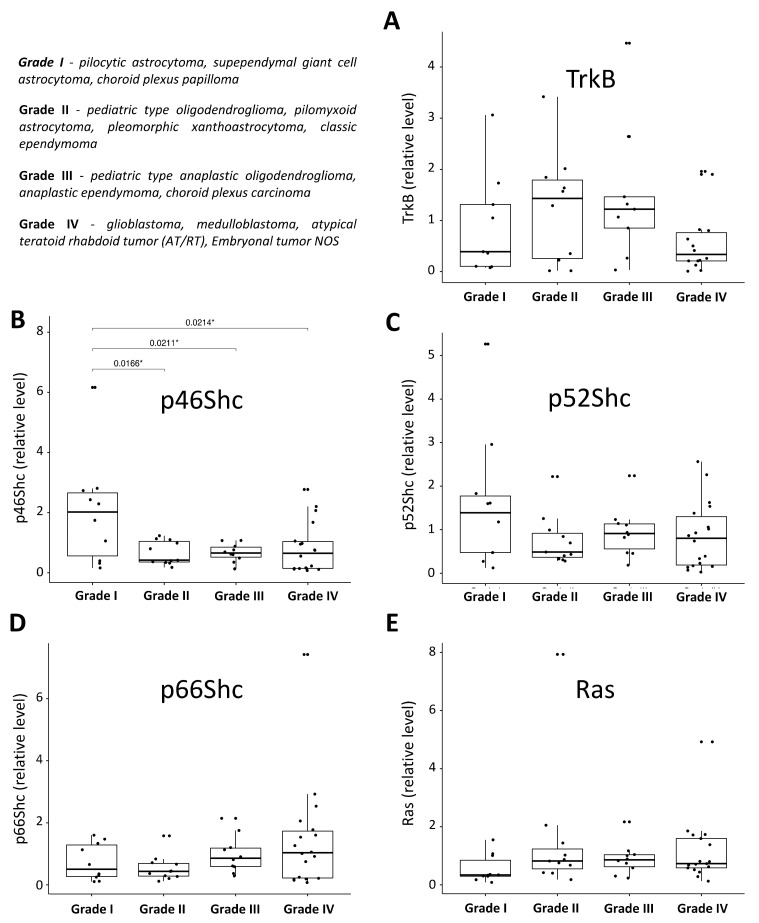
Box plot graphs showing the TrkB, Ras, and ShcA isoforms’ western blot quantification in the investigated pediatric brain tumors divided by the grade of malignancy. The presented box plots show the standardized levels of TrkB (**A**), p66Shc (**D**), p52Shc (**C**), p46Shc (**B**), and Ras (**E**) proteins in the four groups of tumor samples divided based on the WHO grade, representing the severity and prognosis of the condition. The assignment of the grade to the tumor type is shown in the top left corner. The position of each dot on the vertical axis indicates values for an individual data point. * *p* < 0.05. The level of TrkB, p66Shc, p52Shc, p46Shc, and Ras was assessed by the western blot technique with the use of specific antibodies and subjected to the statistical analysis described in the Materials and Methods section.

Densitometric analysis of the western blot bands allowed us to create an expression pattern of the investigated proteins in the studied pediatric brain tumors samples (Figure 8) and to correlate their levels with the tumor malignancy grade (Figure 9). For each protein (p66Shc, p52Shc, p46Shc, Ras, and TrkB), we performed one-way ANOVA statistical tests and post-hoc t-tests with Bonferroni correction separately for each group of investigated tumors and their malignancy grades. Interestingly, the parametric tests revealed statistically significant differences between CNS embryonal (Group 3) and ependymal (Group 4) tumors for the TrkB protein (Figure 8A). Moreover, we found statistically significant differences (*p* < 0.05) in the level of p46Shc protein between grade I and other grades of malignancy (Figure 9B). The respective nonparametric tests for the grades confirmed this result.

## 4. Discussion

**Figure 10:**
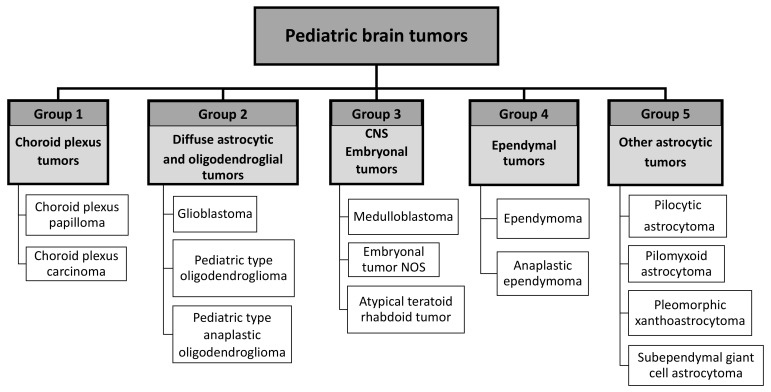
A diagram showing the division of examined pediatric brain tumor groups. Based on the histochemical staining and histopathological assessment, the tumor samples tested for ShcA, Ras, and TrkB proteins in this study were classified into the groups shown in the presented chart and named according to the newest WHO indications. This picture intends to help analyze the data and interpret the results of the experiments presented in the study.

To evaluate the potential correlation between tumor type, tumor malignancy grade, and the expression patterns of Ras, TrkB, and ShcA proteins, we analyzed 49 samples of pediatric brain tumors. In our studies, five main groups were distinguished (Figure 10).

Group 1 was represented by two types of choroid plexus tumors: choroid plexus papilloma, (WHO grade I) and choroid plexus carcinoma (WHO grade III).

Group 2 was represented by three types of diffuse astrocytic and oligodendroglial tumors: (a) glioblastoma (WHO grade IV), (b) pediatric-type oligodendroglioma (WHO grade II), and (c) pediatric-type anaplastic oligodendroglioma (WHO grade III).

Group 3 was represented by CNS embryonal tumors. In this group, we distinguished three types of embryonal tumors: (a) medulloblastoma (WHO grade IV), (b) embryonal tumors not otherwise specified (WHO grade IV), and (c) atypical teratoid/rhabdoid tumors (WHO grade IV).

Group 4 was represented by ependymal tumors. In the range of this group, we distinguished two types of ependymal tumors: (a) conventional ependymoma (WHO grade II) and (b) anaplastic ependymoma (WHO grade III).

Group 5 was represented by tumors characterized by the WHO as other astrocytic tumors. In the range of this group, we distinguished four types of tumors: (a) pilocytic astrocytoma (WHO grade I), (b) pilomyxoid astrocytoma (WHO grade II), (c) pleomorphic xanthoastrocytoma (WHO grade II), and (d) subependymal giant cell astrocytoma (WHO grade I).

Correct assignment of individual samples to each group was confirmed based on histopathological and immunohistochemical investigations.

### 4.1. Expression Pattern of ShcA, Ras, and TrkB Proteins in Studied Pediatric Brain Tumors

Next, we evaluated the expression patterns of ShcA isoforms as well as Ras and TrkB in all characterized tumors represented by the following groups: choroid plexus tumors, diffuse astrocytic and oligodendroglial tumors, embryonal tumors, ependymal tumors, and other astrocytic tumors (see Figure 10 and Supplementary Table S1). It has been previously demonstrated that Ras activity depends on ShcA recruitment upon Trk activation by, for example, neurothrophins [8,46]. Affected functioning of proteins from this pathway may be strictly associated with oncogenic processes, especially because Ras has been described as an oncogene, and its activation may influence the activity of downstream kinases implicated in cell proliferation. Moreover, the Shc upstream molecules such as Trk receptors have been previously correlated with tumor malignancy and have been suggested as a prognostic factor in brain tumors [47,48]. TrkB, in particular, has been reported as a negative prognostic marker in neuroblastoma [48,49].

Analysis of the proteins of interest levels with the use of western blot revealed that the level of Ras protein was comparable in all studied types of brain tumors (Figure 8E). This result may be in line with the actual knowledge about Ras in which the genotype (mutation status) is the main culprit for the different transforming potentials of Ras proteins [50] and tumor aggressiveness. Interestingly, as shown in Figure 8B,C, the levels of two ShcA isoforms (evaluated with the use of specific antibodies) that activate Ras protein, p46Shc and p52Shc, also seem to be equal in all groups. Despite no statistically significant differences, the lowest levels of p46Shc and p52Shc were detected in diffuse astrocytic and oligodendroglial tumors (group 2) and in tumors characterized by the WHO as other astrocytic tumors (group 5) in the case of p52Shc. Similarly, the level of the longest ShcA isoform, p66Shc, did not differ much in the five groups of investigated pediatric brain tumors (Figure 8D). Interestingly, differences in the expression pattern were observed only for the TrkB protein. One-way ANOVA and post-hoc t-tests with Bonferroni correction showed a significant (at the 5% level) difference in the level of TrkB between CNS embryonal tumors (group 3) and ependymal tumors (group 4).

### 4.2. Pediatric Brain Tumor Malignancy Grade and the Pattern of ShcA, Ras, and TrkB Proteins

Additionally, we analyzed whether the protein patterns of Ras, TrkB, and all three isoforms of ShcA protein (p66Shc, p52Shc, and p46Shc) correlate with malignancy grade of the investigated tumors. Importantly, classifying and grading tumors enables the determination of treatment recommendations and prognosis. The simplest classification discriminates two groups: low grade and high grade neoplasms tumors. Low grade tumors are typically slow-growing and rarely spread via cerebrospinal fluid (CSF). They often have well-defined borders, so surgical removal in these cases can be an effective treatment. In contrast, malignant tumors tend to grow faster, and often relapse. The WHO classification of CNS tumors (2016) traditionally comprises a histologic grading to a four-tiered scheme of malignancy ranging from WHO grade I (benign) to WHO grade IV (malignant) lesions. Childhood brain tumors are the most common pediatric solid tumors and include several histological subtypes [51]. Pilocytic astrocytoma is a slowly growing, well-circumscribed, and frequently cystic astrocytoma of children and young adults corresponding to WHO grade I is the most common pediatric tumors of CNS [52]. The most common malignant brain tumor in children is medulloblastoma (WHO grade IV) [53]. Classic medulloblastoma is the most common variant, accounting for up to 70% of cases [53,54]. Another variant of MB such a desmoplastic/nodular medulloblastoma comprises 10–20% of cases [45,53,54]. Large cell/anaplastic (LCA) medulloblastoma comprises 5% of cases and is characterized by very aggressive course [45]. Medulloblastoma with extensive nodularity (MBEN) occurs in infants and presents an extreme degree of desmoplastic/nodular pattern. It likely has a better prognosis due to the degree of neuronal differentiation [45]. Brain tumors corresponding to grade I lesions are neoplasms with low proliferative potential. Grade II lesions are usually infiltrative and often recur with a tendency to progress to higher grades of malignancy. Grade III brain tumors disclose histological features of malignancy including nuclear atypia and mitotic activity. The grade IV designation is applied to mitotically active, necrosis-prone neoplasms with rapid evolution and fatal outcomes. Glioblastoma and embryonal tumors are examples of grade IV neoplasms [45].

Analysis of the TrkB, Ras, and ShcA isoform levels presented in a function of malignancy grade (Figure 9) revealed that only one isoform of ShcA proteins, p46Shc, was significantly elevated in tumors with grade I malignancy in comparison to the other tumors characterized by grades II, III, and IV of malignancy (Figure 9B). A similar trend was observed for the p52Shc protein and malignancy grade, but here the differences were not statistically significant. In contrast to the studies describing an increased level of p66Shc in highly metastatic variants of the human breast cancer cell line MDA-MB-231 or the studies suggesting that decreased levels of p66Shc can be good markers for the diagnosis and prognosis of breast cancer, here in the case of pediatric brain tumors, we did not see such correlations. The level of p66Shc seemed to be equal in all groups of pediatric brain tumors characterized by different malignancy grade (Figure 9D). The Ras level (Figure 9E) also seemed to be equal in tumors with grades I, II, III, and IV of malignancy, indicating that in tumors of grade I, Ras was expressed to the lowest extent (however, this observation was also not statistically significant). These results may be perceived as contradictory to the studies investigating Ras level in tumors of the adult human central nervous system published by Gutierrez-Erlandsson and colleagues [55]. They demonstrated that R-RAS2 is more strongly expressed in low grade (grades I–II) rather than high grade (grades III–IV) tumors of the adult human central nervous system, suggesting that R-RAS2 is overexpressed in the early stages of malignancy. The contradictory results and opposite conclusion regarding Ras level in tumors of the central nervous system can result from two main reasons: (a) in our studies, expression pattern of proteins of interest (including Ras) as investigated in pediatric brain tumors, which have different molecular characteristics compared to the tumors of the adult human central nervous system described by Gutierrez-Erlandsson et al.; and (b) our data reflect the total Ras isoform pool, and in the case of Gutierrez-Erlandsson et al., the dependency on tumor malignancy was described for R-RAS2 only [55]. Additionally, in the case of TrkB, it was difficult to find any linear correlation between its level and malignancy grade. An opposite conclusion arose from the comprehensive meta-analysis by Zhang and coworkers [44], where the expression level of TrkB was strongly and positively associated with the clinical stage (I–II versus III–IV). Based on their analysis, Zhang and coworkers concluded that TrkB can be a potential biomarker for poor prognosis. It is necessary to highlight that such a conclusion was made based on the analysis of a cohort of cancers including gastric [34,36], colorectal [37,38], non-small cell lung [39], and ovarian [40] cancers, nasopharyngeal [41], sinonasal, and oral squamous cell [42,43], and hepatocellular carcinoma [44]. In contrast to the studies described above, our analysis was performed in a cohort of pediatric brain tumors, which were not taken into consideration by Zhang and coworkers [44]. Our study demonstrates that the reliance between the expression level of TrkB and tumor malignancy, described for other solid tumors, differs from that observed for pediatric brain tumors.

## 5. Conclusions

Based on the results of our comprehensive and comparative study, we demonstrated that the expression pattern of proteins such as Ras, ShcA (p66Shc, p52Shc, p46Shc) in astrocytic, oligodendrioglial, ependymal, choroid plexus, and embryonal tumors in children seem to be closely similar. In the case of TrkB, significant differences in its level have been found between CNS embryonal and ependymal tumors. Interestingly, a significantly higher level of p46Shc protein was observed in pediatric brain tumors with malignancy grade I in comparison to the tumors with grades II, III, and IV of malignancy. Such observations indicate that p46Shc and TrkB might be considered as useful biomarkers in the diagnosis and for the prognosis of pediatric brain tumors.

## Figures and Tables

**Table 1:** The list of antibodies used in the immunohistochemical studies.

Antibody	Clone	Host	Supplier
EMA	E29	Mouse	VENATANA/ROCHE
Vimentin	Vim 3B4	Mouse	VENATANA/ROCHE
Pancytokeratin	AE1/AE3 & PCK26	Mouse	VENATANA/ROCHE
Glial Fibrillary Acidic Protein (GFAP)	EP672Y	Rabbit	CELL MARQUE
Neurofilament	2F11	Mouse	CELL MARQUE
Ki67	30.9	Rabbit	VENATANA/ROCHE
Olig2	EP356	Rabbit	CELL MARQUE
S-100	Polyclonal	Rabbit	VENATANA/ROCHE
Synaptophysin	MRQ-40	Rabbit	CELL MARQUE
INI-1	MRQ-27	Mouse	CELL MARQUE

## Data Availability

The authors agree to make the data and materials supporting the results or analyses presented in their paper available upon reasonable request.

## Supplementary Materials

The following are available online at https://www.mdpi.com/article/10.3390/jcm10102219/s1, Table S1: Tumor type, WHO grade, localization, age, year of diagnosis, gender of pediatric brain tumors donors, Table S2: Cytoplasmic expression of GFAP and Ki-67 labeling index in investigated pediatric brain tumors.

## References

[1] Pelicci G., Dente L., de Giuseppe A., Verducci-Galletti B., Giuli S., Mele S., Vetriani C., Giorgio M., Pandolfi P.P., Cesareni G. (1996). A family of Shc related proteins with conserved PTB, CH1 and SH2 regions. Oncogene.

[2] Simanshu D.K., Nissley D.V., Mccormick F. (2017). RAS Proteins and Their Regulators in Human Disease. Cell.

[3] Bhat S.S., Anand D., Khanday F.A. (2015). p66Shc as a switch in bringing about contrasting responses in cell growth: Implications on cell proliferation and apoptosis. Mol. Cancer.

[4] Arias-Romero L.E., Villamar-Cruz O., Pacheco A., Kosoff R., Huang M., Muthuswamy S.K., Chernoff J. (2010). A Rac-Pak signaling pathway is essential for ErbB2-mediated transformation of human breast epithelial cancer cells. Oncogene.

[5] Samuel M.S., Lourenço F.C., Olson M.F. (2011). K-Ras mediated murine epidermal tumorigenesis is dependent upon and associated with elevated Rac1 activity. PLoS ONE.

[6] Menges C.W., Sementino E., Talarchek J., Xu J., Chernoff J., Peterson J.R., Testa J.R. (2012). Group I p21-activated kinases (PAKs) promote tumor cell proliferation and survival through the AKT1 and Raf-MAPK pathways. Mol. Cancer Res..

[7] Ravichandran K.S. (2001). Signaling via Shc family adapter proteins. Oncogene.

[8] Lotti L.V., Lanfrancone L., Migliaccio E., Zompetta C., Pelicci G., Salcini A.E., Falini B., Pelicci P.G., Torrisi M.R. (1996). Shc proteins are localized on endoplasmic reticulum membranes and are redistributed after tyrosine kinase receptor activation. Mol. Cell. Biol..

[9] Migliaccio E., Mele S., Salcini A.E., Pelicci G., Venus Lai K.-M., Superti-Furga G., Pawson T., di Fiore P.P., Lanfrancone L., Pelicci P.G. (1997). Opposite effects of the p52shc/p46shc and p66shc splicing isoforms on the EGF receptor-MAP kinase-fos signalling pathway. EMBO J..

[10] Migliaccio E., Giorgio M., Mele S., Pelicci G., Reboldi P., Pandolfi P.P., Lanfrancone L., Pelicci P.G. (1999). The p66shc adaptor protein controls oxidative stress response and life span in mammals. Nature.

[11] Cheng Y.S., Chao J., Chen C., Lv L.L., Han Y.C., Liu B.C. (2019). The PKCβ-p66shc-NADPH oxidase pathway plays a crucial role in diabetic nephropathy. J. Pharm. Pharmacol..

[12] Boengler K., Bornbaum J., Schlüter K.D., Schulz R. (2019). P66shc and its role in ischemic cardiovascular diseases. Basic Res. Cardiol..

[13] Savino C., Pelicci P., Giorgio M. (2013). The P66Shc/mitochondrial permeability transition pore pathway determines neurodegeneration. Oxid. Med. Cell. Longev..

[14] Lebiedzinska M., Karkucinska-Wieckowska A., Giorgi C., Karczmarewicz E., Pronicka E., Pinton P., Duszynski J., Pronicki M., Wieckowski M.R. (2010). Oxidative stress-dependent p66Shc phosphorylation in skin fibroblasts of children with mitochondrial disorders. Biochim. Biophys. Acta.

[15] Lebiedzinska M., Duszynski J., Rizzuto R., Pinton P., Wieckowski M.R. (2009). Age-related changes in levels of p66Shc and serine 36-phosphorylated p66Shc in organs and mouse tissues. Arch. Biochem. Biophys..

[16] Veeramani S., Yuan T.C., Lin F.F., Lin M.F. (2008). Mitochondrial redox signaling by p66Shc is involved in regulating androgenic growth stimulation of human prostate cancer cells. Oncogene.

[17] Lee M.S., Igawa T., Chen S.J., Van Bemmel D., Lin J.S., Lin F.F., Johansson S.L., Christman J.K., Lin M. (2004). p66Shc protein is upregulated by steroid hormones in hormone-sensitive cancer cells and in primary prostate carcinomas. Int. J. Cancer.

[18] Rajendran M., Thomes P., Zhang L., Veeramani S., Lin M.F. (2010). p66Shc—A longevity redox protein in human prostate cancer progression and metastasis: p66Shc in cancer progression and metastasis. Cancer Metastasis Rev..

[19] Alam S.M., Rajendran M., Ouyang S., Veeramani S., Zhang L., Lin M.F. (2009). A novel role of Shc adaptor proteins in steroid hormone-regulated cancers. Endocr. Relat. Cancer.

[20] Ma Z., Liu Z., Wu R.F., Terada L.S. (2010). p66(Shc) restrains Ras hyperactivation and suppresses metastatic behavior. Oncogene.

[21] Pelicci G., Lanfrancone L., Salcini A.E., Romano A., Mele S., Grazia Borrello M., Segatto O., Di Fiore P.P., Pelicci P.G. (1995). Constitutive phosphorylation of Shc proteins in human tumors. Oncogene.

[22] Cox A.D., Der C.J. (2010). Ras history: The saga continues. Small GTPases.

[23] Milburn M.V., Tong L., Devos A.M., Brünger A., Yamaizumi Z., Nishimura S., Kim S.H. (1990). Molecular switch for signal transduction: Structural differences between active and inactive forms of protooncogenic ras proteins. Science.

[24] Lim K.H., Counter C.M. (2005). Reduction in the requirement of oncogenic Ras signaling to activation of PI3K/AKT pathway during tumor maintenance. Cancer Cell.

[25] Forbes S.A., Beare D., Boutselakis H., Bamford S., Bindal N., Tate J., Cole C.G., Ward S., Dawson E., Ponting L. (2017). COSMIC: Somatic cancer genetics at high-resolution. Nucleic Acids Res..

[26] Bos J.L. (1989). Ras oncogenes in human cancer: A review. Cancer Res..

[27] Davol P.A., Bagdasaryan R., Elfenbein G.J., Maizel A.L., Frackelton A.R.J.R. (2003). Shc proteins are strong, independent prognostic markers for both node-negative and node-positive primary breast cancer. Cancer Res..

[28] Frackelton A.R.J.R., Lu L., Davol P.A., Bagdasaryan R., Hafer L.J., Sgroi D.C. (2006). p66 Shc and tyrosine-phosphorylated Shc in primary breast tumors identify patients likely to relapse despite tamoxifen therapy. Breast Cancer Res..

[29] Grossman S.R., Lyle S., Resnick M.B., Sabo E., Lis R.T., Rosinha E., Liu Q., Hsieh C.-H., Bhat G., Frackelton A.R. (2007). p66 Shc tumor levels show a strong prognostic correlation with disease outcome in stage IIA colon cancer. Clin. Cancer Res..

[30] Jackson J.G., Yoneda T., Clark G.M., Yee D. (2000). Elevated levels of p66 Shc are found in breast cancer cell lines and primary tumors with high metastatic potential. Clin. Cancer Res..

[31] Haines E., Saucier C., Claing A. (2014). The adaptor proteins p66Shc and Grb2 regulate the activation of the GTPases ARF1 and ARF6 in invasive breast cancer cells. J. Biol. Chem..

[32] Sakai R., Henderson J.T., O’bryan J.P., Elia A.J., Saxton T.M., Pawson T. (2000). The mammalian ShcB and ShcC phosphotyrosine docking proteins function in the maturation of sensory and sympathetic neurons. Neuron.

[33] Conti L., Sipione S., Magrassi L., Bonfanti L., Rigamonti D., Pettirossi V., Peschanski M., Haddad B., Pelicci P., Milanesi G. (2001). Shc signaling in differentiating neural progenitor cells. Nat. Neurosci..

[34] Tanaka K., Shimura T., Kitajima T., Kondo S., Ide S., Okugawa Y., Saigusa S., Toiyama Y., Inoue Y., Araki K.T. (2014). Tropomyosin-related receptor kinase B at the invasive front and tumour cell dedifferentiation in gastric cancer. Br. J. Cancer.

[35] Okugawa Y., Tanaka K., Inoue Y., Kawamura M., Kawamoto A., Hiro J., Saigusa S., Toiyama Y., Ohi M., Uchida K. (2013). Brain-derived neurotrophic factor/tropomyosin-related kinase B pathway in gastric cancer. Br. J. Cancer.

[36] Zhang Y., Fujiwara Y., Doki Y., Takiguchi S., Yasuda T., Miyata H., Yamazaki M., Ngan C.Y., Yamamoto H., Ma Q. (2008). Overexpression of tyrosine kinase B protein as a predictor for distant metastases and prognosis in gastric carcinoma. Oncology.

[37] Fan M., Sun J., Wang W., Fan J., Wang L., Zhang X., Yang A., Wang W., Zhang R., Li J. (2014). Tropomyosin-related kinase B promotes distant metastasis of colorectal cancer through protein kinase B-mediated anoikis suppression and correlates with poor prognosis. Apoptosis.

[38] Dawson H., Grundmann S., Koelzer V.H., Galván J.A., Kirsch R., Karamitopoulou E., Lugli A., Inderbitzin D., Zlobec I. (2015). Tyrosine kinase receptor B (TrkB) expression in colorectal cancers highlights anoikis resistance as a survival mechanism of tumour budding cells. Histopathology.

[39] Okamura K., Harada T., Wang S., Ijichi K., Furuyama K., Koga T., Okamoto T., Takayama K., Yano T., Nakanishi Y. (2012). Expression of TrkB and BDNF is associated with poor prognosis in non-small cell lung cancer. Lung Cancer.

[40] Au C.W., Siu M.K., Liao X., Wong E.S., Ngan H.Y., Tam K.F., Chan D.C.W., Chan Q.K.Y., Cheung A.N.Y. (2009). Tyrosine kinase B receptor and BDNF expression in ovarian cancers—Effect on cell migration, angiogenesis and clinical outcome. Cancer Lett..

[41] Li S.S., Liu J.J., Wang S., Tang Q.L., Liu B.B., Yang X.M. (2014). Clinical significance of TrkB expression in nasopharyngeal carcinoma. Oncol. Rep..

[42] Sasahira T., Ueda N., Yamamoto K., Bhawal U.K., Kurihara M., Kirita T., Kuniyasu H. (2013). Trks are novel oncogenes involved in the induction of neovascularization, tumor progression, and nodal metastasis in oral squamous cell carcinoma. Clin. Exp. Metastasis.

[43] Li L., Zhu L. (2017). Expression and clinical significance of TrkB in sinonasal squamous cell carcinoma: A pilot study. Int. J. Oral. Maxillofac. Surg..

[44] Zhang C., Li X., Gao D., Ruan H., Lin Z., Li X., Liu G., Ma Z., Li X. (2017). The prognostic value of over-expressed TrkB in solid tumors: A systematic review and meta-analysis. Oncotarget.

[45] Louis D.N., Perry A., Reifenberger G., Von Deimling A., Figarella-Branger D., Cavenee W.K., Ohgaki H., Wiestler O.D., Kleihues P., Ellison D.W. (2016). The 2016 World Health Organization Classification of Tumors of the Central Nervous System: A summary. Acta Neuropathol..

[46] Zampieri N., Chao M.V. (2006). Mechanisms of neurotrophin receptor signalling. Biochem. Soc. Trans..

[47] Terui E., Matsunaga T., Yoshida H., Kouchi K., Kuroda H., Hishiki T., Saito T., Yamada S.-I., Shirasawa H., Ohnuma N. (2005). Shc family expression in neuroblastoma: High expression of shcC is associated with a poor prognosis in advanced neuroblastoma. Clin. Cancer Res..

[48] Nakagawara A., Arima-Nakagawara M., Scavarda N.J., Azar C.G., Cantor A.B., Brodeur G.M. (1993). Association between high levels of expression of the TRK gene and favorable outcome in human neuroblastoma. N. Engl. J. Med..

[49] Jaboin J., Kim C.J., Kaplan D.R., Thiele C.J. (2002). Brain-derived neurotrophic factor activation of TrkB protects neuroblastoma cells from chemotherapy-induced apoptosis via phosphatidylinositol 3’-kinase pathway. Cancer Res..

[50] Muñoz-Maldonado C., Zimmer Y., Medová M. (2019). A Comparative Analysis of Individual RAS Mutations in Cancer Biology. Front. Oncol..

[51] Pollack I.F., Agnihotri S., Broniscer A. (2019). Childhood brain tumors: Current management, biological insights, and future directions. J. Neurosurg. Pediatr..

[52] Salles D., Laviola G., Malinverni A.C.M., Stavale J.N. (2020). Pilocytic Astrocytoma: A Review of General, Clinical, and Molecular Characteristics. J. Child Neurol..

[53] Kline C.N., Packer R.J., Hwang E.I., Raleigh D.R., Braunstein S., Raffel C., Bandopadhayay P., Solomon D.A., Aboian M., Cha S. (2017). Case-based review: Pediatric medulloblastoma. Neurooncol. Pract..

[54] Juraschka K., Taylor M.D. (2019). Medulloblastoma in the age of molecular subgroups: A review. J. Neurosurg. Pediatr..

[55] Gutierrez-Erlandsson S., Herrero-Vidal P., Fernandez-Alfara M., Hernandez-Garcia S., Gonzalo-Flores S., Mudarra-Rubio A., Fresno M., Cubelos B. (2013). R-RAS2 overexpression in tumors of the human central nervous system. Mol. Cancer.

