# Supplementary material for: Ras, TrkB, and ShcA Protein Expression Patterns in Pediatric Brain Tumors
Source: J Clin Med. 2021 May 20;10(10):2219. doi: 10.3390/jcm10102219 (PMC8160917; doi:10.3390/jcm10102219)
Supplement: Supplementary file 1 [file jcm-10-02219-s001.zip › jcm-1141139-supplementary.pdf]

Supplementary Table 1. Tumor type, WHO grade, localization, age, year of diagnosis, gender of pediatric brain tumors donors (part 1)

	Pediatric brain tumors	WHO grade	Localization	Age (Y)	Sex	Year of diagnosis
Group 1	Choroid plexus tumors					
	choroid plexus papilloma (CPP)	G I	fourth ventricle	16	F	2010
	choroid plexus papilloma (CPP)	G I	lateral ventricle	9	M	2012
	choroid plexus papilloma (CPP)	G I	lateral ventricle	1	F	2012
	choroid plexus carcinoma (CPC)	G III	lateral and fourth ventricles	3	F	2014
	choroid plexus carcinoma (CPC)	G III	lateral ventricle	9	M	2014
	choroid plexus carcinoma (CPC)	G III	lateral and fourth ventricles	1	M	2012
Group 2	Diffuse astrocytic and oligodendroglial tumors					
	Glioblastoma (GB)	G IV	temporal lobe	11	F	2009
	Glioblastoma (GB)	G IV	temporal lobe	11	F	2011
	Glioblastoma (GB)	G IV	temporal lobe	6	M	2013
	pediatric type oligodendroglioma	G II	thalamus	13	M	2014
	pediatric type oligodendroglioma	G II	hippocampus	2	M	2013
	pediatric type oligodendroglioma	G II	temporal lobe	1	M	2011
	pediatric type oligodendroglioma	G II	parietal lobe	10	M	2011
	pediatric type anaplastic oligodendroglioma	G III	occipital lobe	3	F	2012
	pediatric type anaplastic oligodendroglioma	G III	occipital lobe	12	F	2011
	pediatric type anaplastic oligodendroglioma	G III	occipital lobe	5	F	2011
Group 3	Embryonal tumors					
	medulloblastoma classic type (MB)	G IV	fourth ventricle	15	F	2012
	medulloblastoma classic type (MB)	G IV	fourth ventricle	5	M	2013
	medulloblastoma classic type (MB)	G IV	posterior fossa	6	M	2012
	medulloblastoma classic type (MB)	G IV	posterior fossa	6	M	2013
	medulloblastoma classic type (MB)	G IV	posterior fossa	7	F	2014
	medulloblastoma classic type (MB)	G IV	fourth ventricle	4	M	2012
	medulloblastoma classic type (MB)	G IV	fourth ventricle	13	M	2012
	medulloblastoma classic type (MB)	G IV	fourth ventricle	12	M	2009
	medulloblastoma classic type (MB)	G IV	posterior fossa	2	F	2013
	medulloblastoma classic type (MB)	G IV	fourth ventricle	2	F	2010
	embryonal tumor NOS	G IV	third ventricle	4	F	2014
	embryonal tumor NOS	G IV	frontal lobe	4	F	2013
	embryonal tumor NOS	G IV	parietal lobe	4	M	2009
	atypical teratoid rhabdoid tumor (ATRT)	G IV	temporal, parietal and occipital lobes	1	M	2014
	atypical teratoid rhabdoid tumor (ATRT)	G IV	posterior fossa	5	F	2010
	atypical teratoid/rhabdoid tumor (ATRT)	G IV	posterior fossa	1	F	2010

Supplementary Table 1. Tumor type, WHO grade, localization, age, year of diagnosis, gender of pediatric brain tumors donors (part 2)

Pediatric brain tumors		WHO grade	Localization	Age	Sex	Year of diagnosis
Group 4	Ependymal tumors					
	ependymoma	G II	posterior fossa	1	M	2012
	ependymoma	G II	posterior fossa	2	F	2012
	anaplastic ependymoma	G III	posterior fossa	1	M	2013
	anaplastic ependymoma	G III	posterior fossa	9	F	2010
	anaplastic ependymoma	G III	posterior fossa	5	M	2013
	anaplastic ependymoma	G III	posterior fossa	4	F	2012
Group 5	Other astrocytic tumors					
	pilocytic astrocytoma	G I	cerebellum	14	F	2013
	pilocytic astrocytoma	G I	cerebellum	3	M	2013
	pilocytic astrocytoma	G I	cerebellum	17	F	2011
	pilocytic astrocytoma	G I	cerebellum	4	M	2011
	pilomyxoid astrocytoma	G II	third ventricle	2	M	2010
	pilomyxoid astrocytoma	G II	third ventricle	5	M	2011
	pilomyxoid astrocytoma	G II	third ventricle	4	F	2011
	pleomorphic xanthoastrocytoma (PXA)	G II	temporal lobe	9	M	2009
	pleomorphic xanthoastrocytoma (PXA)	G II	temporal lobe	13	F	2013
	pleomorphic xanthoastrocytoma (PXA)	G II	temporal lobe	17	M	2010
	supependymal giant cell astrocytoma (SEGA)	G I	lateral ventricle	14	M	2013
	supependymal giant cell astrocytoma (SEGA)	G I	lateral ventricle	6	F	2011
	supependymal giant cell astrocytoma (SEGA)	G I	lateral ventricle	7	M	2010

Supplementary Table 2. Cytoplasmic expression of GFAP and Ki-67 labeling index in investigated pediatric brain tumors (part 1)

Pediatric brain tumors		WHO grade	GFAP immunoreactivity	Ki67 LI
Group 1	Choroid plexus tumors			
	choroid plexus papilloma (CPP)	G I	-	1
	choroid plexus papilloma (CPP)	G I	-	1
	choroid plexus papilloma (CPP)	G I	-	1
	choroid plexus carcinoma (CPC)	G III	-	25
	choroid plexus carcinoma (CPC)	G III	-	20
	choroid plexus carcinoma (CPC)	G III	-	20
Group 2	Diffuse astrocytic and oligodendroglial tumors			
	Glioblastoma (GB)	G IV	+++	60
	Glioblastoma (GB)	G IV	+++	55
	Glioblastoma (GB)	G IV	+++	50
	pediatric type oligodendroglioma	G II	_	1
	pediatric type oligodendroglioma	G II	_	2
	pediatric type oligodendroglioma	G II	+	1
	pediatric type oligodendroglioma	G II	-	2
	pediatric type anaplastic oligodendroglioma	G III	++	40
	pediatric type anaplastic oligodendroglioma	G III	-	20
	pediatric type anaplastic oligodendroglioma	G III	-	20
Group 3	Embryonal tumors			
	Medulloblastoma classic type (MB)	G IV	-	50
	medulloblastoma classic type (MB)	G IV	-	50
	medulloblastoma classic type (MB)	G IV	-	50
	medulloblastoma classic type (MB)	G IV	-	50
	medulloblastoma classic type (MB)	G IV	-	70
	Medulloblastoma classic type (MB)	G IV	-	70
	medulloblastoma classic type (MB)	G IV	-	70
	medulloblastoma classic type (MB)	G IV	-	60
	medulloblastoma classic type (MB)	G IV	-	80
	medulloblastoma classic type (MB)	G IV	-	50
	embryonal tumor NOS	G IV	+	75
	embryonal tumor NOS	G IV	-	70
	embryonal tumor NOS	G IV	-	80
	atypical teratoid rhabdoid tumor (ATRT)	G IV	+++	80
	atypical teratoid rhabdoid tumor (ATRT)	G IV	+	75
	atypical teratoid/rhabdoid tumor (ATRT)	G IV	+	75

Cytoplasmic expression of glial fibrillary acid protein (GFAP) was assessed semiquantitatively as negative (-), low (+), moderate (++), and strong (+++), depending on the percentage of positive cells in microscopic examination.

Ki-67 labeling index expressed as percentage of nuclear expression of Ki-67 counted in 300 hot-spot neoplastic cells in high power microscopic fields (400x).

Supplementary Table 2. Cytoplasmic expression of GFAP and Ki-67 labeling index in investigated pediatric brain tumors (part 2)

Pediatric brain tumors		WHO grade	GFAP immunoreactivity	Ki67 LI
Group 4	Ependymal tumors			
	ependymoma	G II	+++	3
	ependymoma	G II	++	3
	anaplastic ependymoma	G III	++	35
	anaplastic ependymoma	G III	++	30
	anaplastic ependymoma	G III	++	30
	anaplastic ependymoma	G III	++	35
Group 5	Other astrocytic tumors			
	pilocytic astrocytoma	G I	+++	3
	pilocytic astrocytoma	G I	+++	2
	pilocytic astrocytoma	G I	+++	2
	pilocytic astrocytoma	G I	+++	3
	pilomyxoid astrocytoma	G II	+++	2
	pilomyxoid astrocytoma	G II	+++	2
	pilomyxoid astrocytoma	G II	+++	2
	pleomorphic xanthoastrocytoma (PXA)	G II	+++	2
	pleomorphic xanthoastrocytoma (PXA)	G II	++	2
	pleomorphic xanthoastrocytoma (PXA)	G II	++	1
	supependymal giant cell astrocytoma (SEGA)	G I	++	1
	supependymal giant cell astrocytoma (SEGA)	G I	++	1
	supependymal giant cell astrocytoma (SEGA)	G I	++	1

Cytoplasmic expression of glial fibrillary acid protein (GFAP) was assessed semiquantitatively as negative (-), low (+), moderate (++), and strong (+++), depending on the percentage of positive cells in microscopic examination.

Ki-67 labeling index expressed as percentage of nuclear expression of Ki-67 counted in 300 hot-spot neoplastic cells in high power microscopic fields (400x).
